# 7T versus 3T phosphorous magnetic resonance spectroscopy in patients with dilated cardiomyopathy

**DOI:** 10.1186/1532-429X-17-S1-P249

**Published:** 2015-02-03

**Authors:** Victoria Stoll, William T Clarke, Eylem Levelt, Saul G Myerson, Matthew D Robson, Stefan Neubauer, Chris Rodgers

**Affiliations:** Division of Cardiovascular Medicine, Radcliffe Department of Medicine, University of Oxford Centre for Clinical Magnetic Resonance Research (OCMR), Oxford, UK

## Background

Phosphorous magnetic resonance spectroscopy (^31^P-MRS) allows insight into cardiac energetics *in vivo*, but it is a technique with an intrinsically low signal to noise ratio (SNR). The 2.4x increased SNR, that is predicted by theory at 7T compared to 3T, should allow detection of smaller changes in metabolite concentrations or measurement of changes in smaller patient groups, which will further our understanding of cardiac energetics. This study represents the first cardiac ^31^P-MRS patient data acquired at a 7 Tesla field strength.

## Methods

10 dilated cardiomyopathy patients were enrolled. All underwent ^31^P-MRS at 3T and 7T field strengths. There were 4 females and 6 males; mean age 57 years (range 36-72). All patients were NYHA Class I (40%) or Class II (60%), and the mean LV ejection fraction was 38%.

## Results

As expected, the change in field strength from 3T to 7T resulted in an increased mean SNR from 7 ± 1.4 to 18 ± 9.3 for PCr (p 0.005 paired t-test) and for γATP an increase from 4.1 ±1.1 to 12.0 ± 3.4 (p 0.0002).The ATP line width increased from 8.9 ±1.7Hz at 3T to 34.1 ± 14.5 at 7T (p 0.0008) a 281% increase, compared to a previously reported increase of 131%. This suggests that more careful B0 shimming may be required in patients than in healthy volunteers.

The PCr/ATP ratio (using all peaks to quantify ATP) was consistent between the two field strengths; with as expected no statistically significant difference, as demonstrated in figure 1a below. At 3T the value was 1.7 ± 0.5 compared to 1.5 ± 0.4 at 7T (p 0.42).

**Figure:**
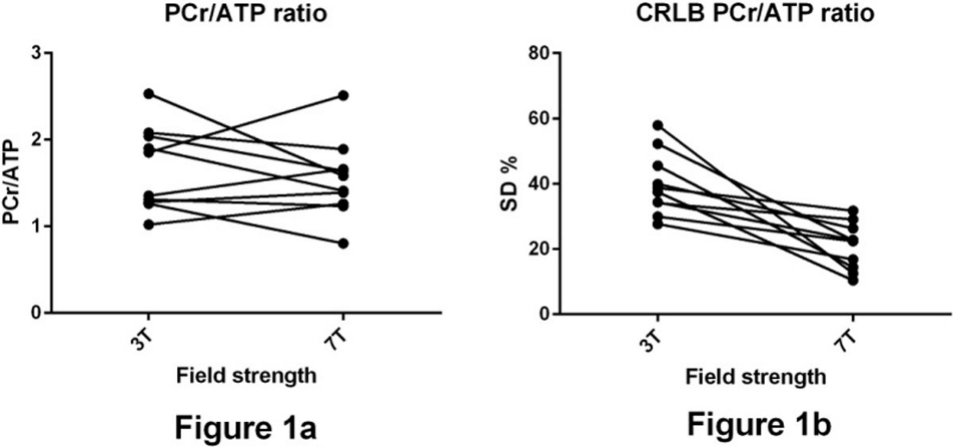
Figure 1

**Figure 2:**
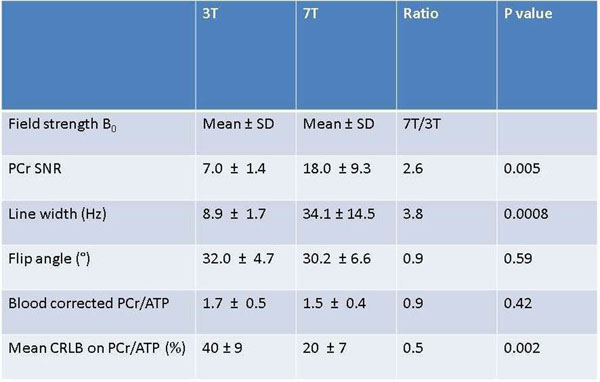
Table showing 3T versus 7T values

The Cramer-Ráo Lower Bounds (CRLB) are shown in figure 1b. The mean PCr/ATP CRLB was 40 ± 9% at 3T compared to 20 ± 7% at 7T (p 0.002). The CRLB gives a lower bound to the accuracy of the fitted parameters. Hence, this decrease represents a desirable increased precision in metabolite quantitation at 7T compared to 3T.

## Conclusions

Compared to a 2.8x SNR increase from 3T to 7T previously reported in normal volunteers, we observed a 2.6x increase in this population of patients. The PCr/ATP ratio is not significantly different between the field strengths, showing there is no significant new bias at 7T. 7T ^31^ P-MRS has been previously shown to be feasible in healthy volunteers and now in patients, which in future should allow more accurate quantification and understanding of myocardial energetics.

## Funding

Dr Victoria Stoll is funded by the British Heart Foundation FS/12/14/29354. The research was supported by a Sir Henry Dale Fellowship from the Wellcome Trust and the Royal Society [Grant Number 098436/Z/12/Z] and by the National Institute for Health Research (NIHR) Oxford Biomedical Research Centre based at The Oxford University Hospitals Trust at the University of Oxford.

